# Creep Resistance and Microstructure Evolution in P23/P91 Welds

**DOI:** 10.3390/ma18010194

**Published:** 2025-01-05

**Authors:** Vlastimil Vodárek, Jan Holešinský, Zdeněk Kuboň, Renáta Palupčíková, Petra Váňová, Jitka Malcharcziková

**Affiliations:** 1 Faculty of Materials Science and Technology, VŠB-Technical University of Ostrava, 17. listopadu 2172/15, 708 00 Ostrava, Czech Republic; renata.palupcikova@vsb.cz (R.P.); petra.vanova@vsb.cz (P.V.); jitka.malcharczikova@vsb.cz (J.M.); 2 CEZ, a.s., Duhová 2/1444, 140 53 Prague, Czech Republic; jan.holesinsky@cez.cz; 3 Material and Metallurgical Research, Ltd., Pohraniční 31, Vítkovice, 706 02 Ostrava, Czech Republic; creep.lab@mmv.cz

**Keywords:** heterogeneous welds P23/P91, creep behavior, fusion zone, microstructural evolution, thermodynamic simulation, kinetic simulation, minor phases

## Abstract

This paper summarizes the results of investigations into heterogeneous P23/P91 welds after long-term creep exposure at temperatures of 500, 550 and 600 °C. Two variants of welds were studied: In Weld A, the filler material corresponded to P91 steel, while in Weld B, the chemical composition of the consumable material matched P23 steel. The creep rupture strength values of Weld A exceeded those of Weld B at all testing temperatures. Most failures in the cross-weld samples occurred in the partially decarburized zones of P23 or WM23 steel. The results of the investigations on the minor phases were in good agreement with kinetic simulations that considered a 0.1 mm fusion zone. Microstructural studies proved that carburization occurred in the P23/P91 weld fusion zones. The partial decarburization of P23 steel or WM23 was accompanied by the dissolution of M_7_C_3_ and M_23_C_6_ particles, and detailed studies revealed the precipitation of the Fe_2_ (W, Mo) Laves phase in decarburized areas. Thermodynamic simulations proved that the appearance of this phase in partially decarburized P23 steel or WM23 is related to a reduction in the carbon content in these areas. According to the results of creep tests, the EBSD investigations revealed a better microstructural stability of the partially decarburized P23 steel in Weld A.

## 1. Introduction

Heterogeneous welds of bainitic 2.5CrMo (W) V and martensitic (9–12)%Cr steels are frequently used in modern thermal power plants to compensate for local differences in temperature, pressure, and corrosive/oxidation environments [1,2,3,4,5]. The microstructural stability of these joints during thermal exposure or creep can be significantly affected by the redistribution of interstitial elements across the fusion boundary. This is driven by the gradients of the activities of these elements, especially carbon [6]. Interstitial atoms move to areas with a lower activity, and the diffusion flux is approximately proportional to the absolute value of the activity difference. Gradual decarburization can cause the weakening of the low-alloy steel zone adjacent to the high-alloy steel. The microstructure gradients in the HAZ are related to the corresponding gradients in the creep properties [7]. The creep characteristics of the individual parts of the welds and their mutual interaction determine the response of the welds to deformation. Loading applied across the welds is expected to cause complex constraint effects in locations where the creep resistance reaches a minimum, and may contribute to local damage. Mismatch in the creep deformation rates exhibited by the base materials, the HAZ, and the weld metals can lead to the accumulation of strain. Loading across the heterogeneous microstructure of the welds plays an important role in the local formation of triaxial stresses and the process of grain boundary sliding [8]. Generally, the criterion of the lowest hardness is not valid to identify the location of creep failure in welds [7]. The accumulation of strain in the narrow zone in the early stages of creep can result in preferential crack nucleation and can accelerate crack growth. Furthermore, residual stresses in welds can accelerate the growth of minor phases, and are therefore regarded as detrimental to creep resistance [9,10].

Heat-resistant steel P/T23 was developed for the manufacture of water walls, reheaters, and superheaters in USC boilers [11]. Modifying the chemical composition of the classical T22 steel grade (2.25Cr-1Mo) by reducing the carbon content and by adding tungsten, vanadium, niobium, boron and nitrogen led to a significant increase in creep resistance [11,12]. A reduced carbon content in P/T23 steel results in a decrease in hardness in the HAZ and cold cracking susceptibility. However, welds made of this steel without post-weld heat treatment (PWHT) were found to be prone to cracking during the early stages of service operation [13]. Long et al. [14] studied cracking in heterogeneous T23/12Cr1MoV welds after about 8000 h of service and found that cracks occurred in the coarse-grained part of the heat-affected zone (CGHAZ) of T23 steel and exhibited typical features of stress-relief cracking (SRC). The SRC phenomenon can be caused by the weakening of grain boundaries due to intensive intragranular precipitation or by impurity segregation [15]. Y. Li and X. Wang [16] studied the evolution of microstructure in simulated CGHAZ in T23 steel during aging at 650 °C and reported the following precipitation sequence: M_3_C → M_3_C + M_7_C_3_ + M_23_C_6_ → M_3_C + M_7_C_3_ + M_23_C_6_ + MX → M_23_C_6_ + MX + M_6_X. During long-term creep service, additional precipitation reactions take place, and these processes can significantly affect the creep resistance of T/P23 welds [17,18,19].

P/T91 steel was developed for applications in USC power plants by modifying 9Cr-1Mo steel with small additions of vanadium, niobium and nitrogen, resulting in nearly doubled creep strength [20,21,22]. This high creep strength is related to the optimized microstructure where the M_23_C_6_-phase particles hinder the formation and growth of subgrains in tempered martensite, while the fine MX (VN) particles in martensitic laths interact with moving dislocations. These minor phases can form during PWHT or during creep exposure. Furthermore, during creep exposure, the Fe_2_Mo Laves-phase particles nucleate in the matrix. Particles of this minor phase exhibit low-dimensional stability. The microstructural stability and creep resistance of (9–12)%Cr martensitic steels can be adversely affected by the gradual replacement of fine VN particles by the modified Z phase (Cr (V, Nb)N), which is the thermodynamically stable nitride in these steels [23,24]. The kinetics of this transition are significantly dependent on the chromium content in the steel [25]. In 9%Cr steels, this transition process is expected to be very slow [26].

Heterogeneous welds where low-alloy steels are joined to high-alloyed ferritic grades are frequently used in advanced thermal power plants. There is no standard approach to the design and fabrication of ferritic–ferritic heterogeneous welds in the power industry. This is closely related to a wide range of allowable stresses for advanced creep-resistant ferritic grades up to approximately 650 °C. There are a number of factors which govern the performance of ferritic heterogeneous welds during creep service: design, fabrication, weldability, composition of the filler materials, PWHT parameters, strength of the filler metal, combinations of base and filler metals, and the end use application [27]. No explicit recommendations exist for filler consumable selection when welding different grades of ferric steels. Some filler material producers provide fabricators with recommendations, but there is no consensus regarding the “best practice”. That is why designers often use different approaches, leading to significant variations in the behavior of these welds.

P23/P91 heterogeneous welds can be used for joining a thick section header made of P91 steel to P23 stubs, or in the production of welded turbine rotors tailored for thermal and mechanical loads at different parts of the turbines. There are at least seven different approaches for the selection of filler metals for ferritic heterogeneous welds [27]. This paper deals with two designs of P23/P91 heterogeneous welds, where the filler consumable matches either to steel grade P91 or P23. The aim of this paper is to compare the creep behavior of these two types of heterogeneous P23/P91 welds and to discuss the results of detailed investigations into the microstructural evolution of the critical parts of these welds during long-term creep exposure at 500, 550 and 600 °C.

## 2. Materials and Methods

**Figure 1:**
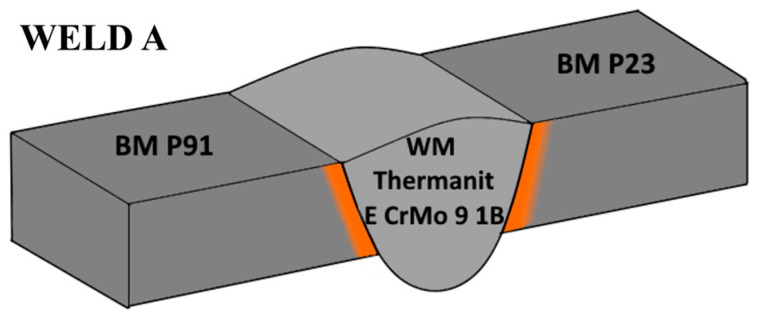
Design of Weld A filler material. Thermanit E CrMo 9 1B matches P91 steel.

**Figure 2:**
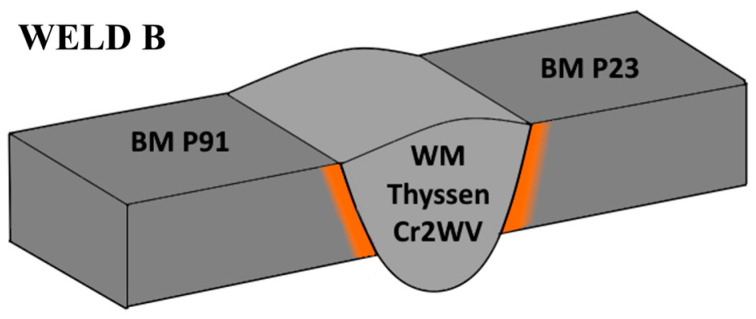
Design of Weld B filler material. Thyssen Cr2WV matches P23 steel.

Hot-rolled steel pipes of ϕ219 × 25 mm made of P91 and P23 steel were used to prepare circumferential weld joints. The quality heat treatment of the P91 steel pipes consisted of normalization at 1050 °C, followed by cooling in air and tempering at 750 °C for 6 h with subsequent cooling in air. The P23 steel pipes were austenitized at 1050 °C, quenched in water, and tempered at 760 °C for 6 h with subsequent cooling in air. Weld A was manufactured using a P91 matching filler metal (Thermanit E CrMo 9 1B), while for Weld B, a consumable matching P23 (Thyssen Cr2WV) was used, as shown in Figure 1 and Figure 2. GTAW (141) technology was applied to produce weld roots, followed by multi-pass welds (7 beads) formed by the SMAW (111) process.

**Figure 3:**
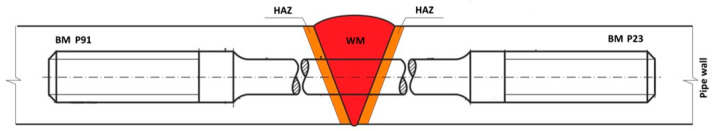
Sketch of the cross-weld creep test sample.

The PWHT regime for both weldments was 750 °C/2 h/air. Table 1 shows the chemical composition of the base materials (BMs) and the weld metals (WMs). Cross-weld creep samples with a diameter in the range from 5 to 8 mm were cut in the middle of the pipe wall thickness (Figure 3). Uniaxial stress rupture tests were performed in air at temperatures of 500, 550 and 600 °C at five stress levels for each temperature.

Longitudinal sections of the creep-ruptured samples were prepared for the microstructure characterization and identification of failure locations using light microscopy (LM). Polished metallographic samples were etched in a V2A solution (a mixture of 10 mL of HNO_3_, 100 mL of HCl and 100 mL of H_2_O) and observed via an Olympus GX51 optical microscope (Olympus Corporation, Tokyo, Japan). Scanning electron microscopy (SEM) was performed using a Quanta 450FEG microscope (Thermo Fischer Scientific, Brno, Czech Republic) equipped with electron backscattered diffraction (EBSD, EDAX Inc., Mahwah, NJ, USA) and X-ray energy-dispersive microanalysis (EDX, EDAX Inc., Mahwah, NJ, USA) facilities. Samples for the EBSD analysis were finally polished on colloidal silica. EBSD mapping was performed at an acceleration voltage of 15 kV and a step size of 0.1 mm. Investigations into the minor phases in creep-ruptured samples were carried out using a JEOL JEM 2100 transmission electron microscope (TEM) equipped with an energy-dispersive (EDX) analyzer (JEOL Ltd., Tokyo, Japan). Both thin foils and carbon-extraction replicas were used. The focused ion beam (FIB, dual-beam FEI NUOVA 600 NANOLAB, Thermo Fischer Scientific, Brno, Czech Republic) technique was used for the preparation of thin foils in the WM23/P91 fusion zone. Carbon extraction replicas were prepared in the following areas of the creep-ruptured cross-weld samples: base materials P23 and P91, weld metals, and the partly decarburized and carburized areas of the welds. The identification of minor phases was performed with a combination of selected area electron diffraction (SAED) and EDX analyses. Interstitial elements were not included in the quantification of the EDX spectra and the results were normalized to 100%. The creep parameters of the samples used for microstructure characterization are summarized in Table 2. Vickers microhardness measurements were carried out on longitudinal metallographic sections of the P23/P91 fusion zones using EmcoTest Durascan 70 G equipment (EmcoTest Prűfmaschinen GmbH, Kuchl, Austria) at a load of 0.01 kg.

Simulations of the thermodynamic equilibrium for the P23 and P91 steels were carried out using Thermo-Calc software 2019b (Thermo-Calc software, Stockholm, Sweden) and the TCFE 8 database. Calculations of minor-phase profiles across the fusion zone of the P23/P91 welds at 600 °C and 30,000 h exposure were performed using Thermo-Calc and Dictra software 2019b. The width of the fusion zone was determined using EDX quantitative data perpendicular to the fusion boundary of the welds.

## 3. Results

### 3.1. Creep Properties

The evaluation of the creep properties of the heterogeneous P23/P91 welds was carried out using the parametric Larson–Miller equation of the first order [29]:

(1) $$P_{LM}=T\cdot \left(C+\log t_{r}\right)$$

*T* is the testing temperature in Kelvin (K), *C* is the constant, and *t_r_* is the time to rupture in hours. The constant *C* = 25 was used.

The creep rupture strength values in 100,000 h (R_u_/_100,000h/T_) were calculated using the Seifert parametric equation in the following form [30]:

(2) $$\log R_{uT}=A_{0}+A_{1}P+A_{2}P^{2},P=\left[T\cdot \left(C+\log t_{r}\right)\cdot 10^{-4}\right]$$

*T* is the testing temperature in Kelvin (K), *t_r_* is the time to rupture in hours and *A*_1_, *A*_2_, *A_k_* and *C* are material constants.

**Figure 4:**
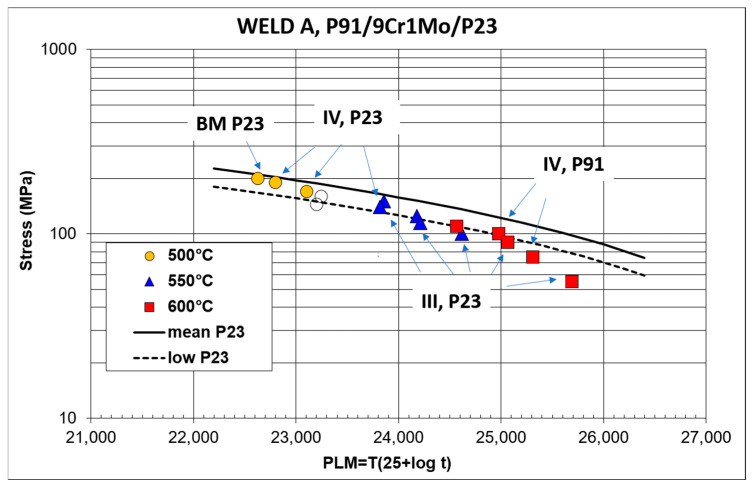
Results of creep rupture tests at 500, 550 and 600 °C and failure locations. Open symbols represent interrupted tests—Weld A.

**Figure 5:**
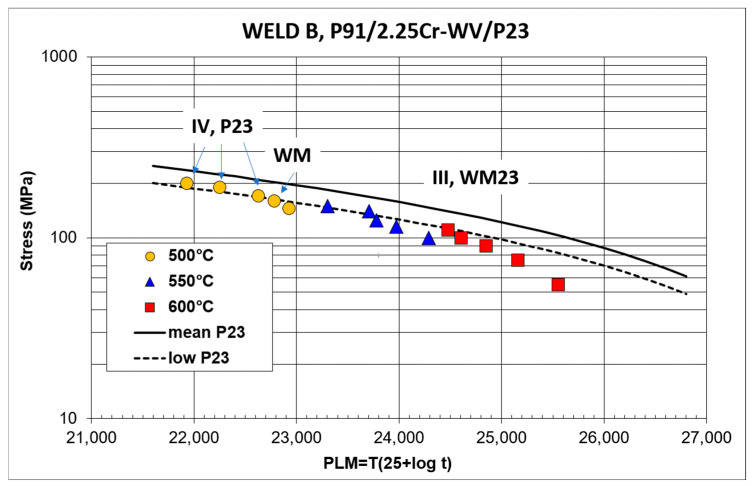
Results of the creep rupture tests at 500, 550 and 600 °C and failure locations—Weld B.

Figure 4 and Figure 5 summarize the results of the stress rupture tests on the cross-weld samples of Welds A and B in the form of the stress dependence of the Larson–Miller parameter. The experimental results are compared with the standardized mean creep strength curve for P23 steel, which exhibits lower creep resistance of both included steels. The dashed curves represent the allowed 20% deviation from the mean creep rupture strength. As is evident, some experimental results fall below the 20% limit of creep rupture strength, especially at a temperature of 600 °C and longer times to rupture. Table 3 shows the results of the calculations of the creep rupture strength of Welds A and B for 100,000 h at temperatures of 500, 550 and 600 °C.

Figure 4 and Figure 5 also include information on failure locations. In the Weld A creep testing, failures were usually reported in the CGHAZ of the P23 steel (type III cracking). At high applied stresses, creep fractures occurred in the intercritical or fine-grained part of the HAZ (ICHAZ, FGHAZ) of the P23 steel (type IV cracking [7]). Creep damage developed simultaneously in several parts of the cross-weld samples, and the final failure occurred in the weakest area. The formation of creep defects was often observed in the HAZ of the P91 steel. Creep tests at 600 °C and applied stresses of 100 and 75 MPa resulted in the final failure in the FGHAZ of the P91 steel (type IV cracking, Figure 4). In the cross-weld samples of Weld B, failures preferentially occurred in WM23 close to the WM23/P91 fusion zone. Only creep samples tested at 500 °C with high applied stresses were an exception. In these samples, creep fractures occurred in the ICHAZ of the P23 steel (Figure 5). In both welds, the creep ductility of the cross-weld samples, which failed in the partly decarburized CGHAZ of the P23 steel or in WM23, reached only a few percent (less than 9%).

### 3.2. Microstructure Characterization of Weld A

**Figure 6:**
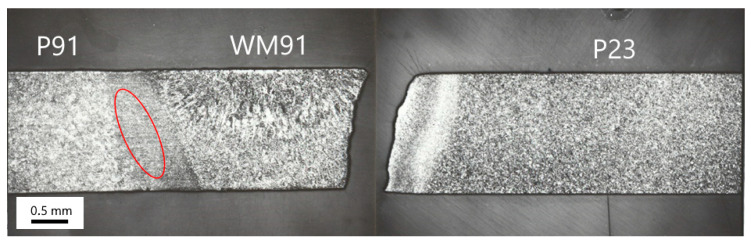
Longitudinal section of the creep-ruptured A1 sample.

**Figure 7:**
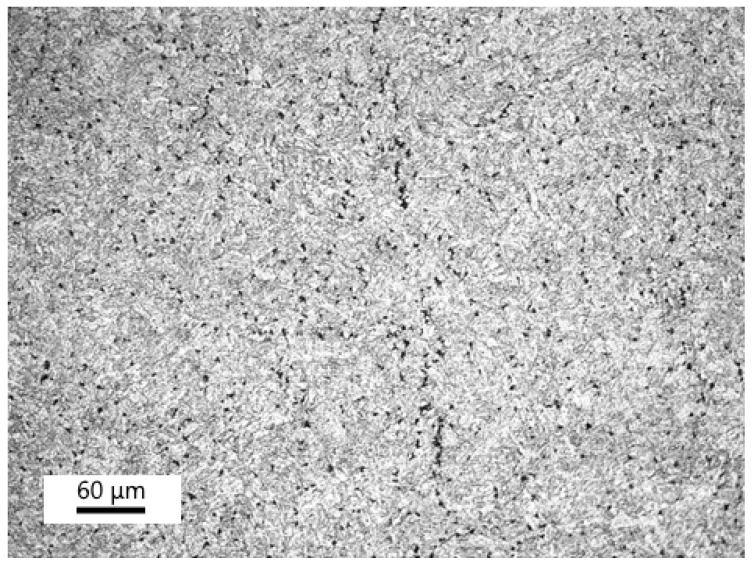
Cavities in the IC/FGHAZ of P91 steel—sample A1.

Figure 6 shows longitudinal sections of the creep-ruptured A1 sample. The creep fracture occurred in the partly decarburized CGHAZ of the P23 steel, close to the fusion boundary. Furthermore, creep damage was also observed in the intercritical and/or fine-grained part of the HAZ on the side of the P91 steel, as shown in the ellipse in Figure 6. Fine cavities in the IC/FGHAZ of the P91 steel are documented in Figure 7. This shows that in Weld A, creep defects simultaneously formed in both HAZs of the cross-weld samples.

**Figure 8:**
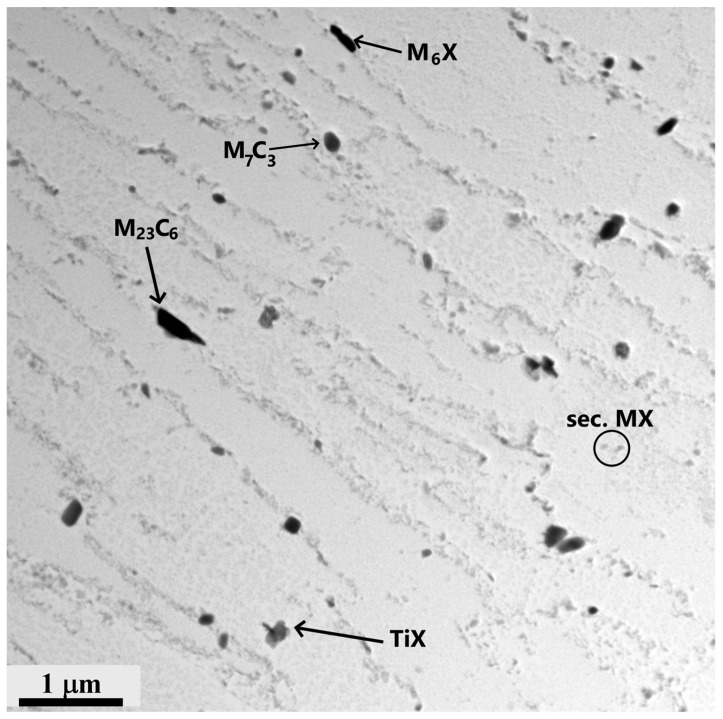
Precipitation of minor phases in the base material P23—sample A2.

The microstructure of the P23 base material after creep exposure corresponded to heavily tempered bainite. Figure 8 shows the typical distribution of the minor phases in the P23 steel in sample A2. M_6_X and M_23_C_6_ particles preferentially nucleated at the prior austenite grain boundaries and lath boundaries. The medium-sized particles were identified as the M_7_C_3_ phase. The chemical composition of these minor phases, as determined by EDX microanalysis, is summarized in Table 4.

Fine particles were formed by the secondary MX phase. The addition of 0.06 wt.% Ti to P23 steel (Table 1) affected the chemical composition, shape, size, and stability of the MX particles. Cubes of titanium-rich, primary MX particles formed during solidification and did not dissolve even at the highest peak temperatures of welding. They were coarser than the secondary vanadium-rich MX particles, which can precipitate during PWHT and/or creep. The fine secondary MX particles that precipitated during creep exposure were significantly enriched with tungsten. The SAED analysis of the particles containing up to about 50 wt.%W confirmed the FCC unit cell of the MX phase.

**Figure 9:**
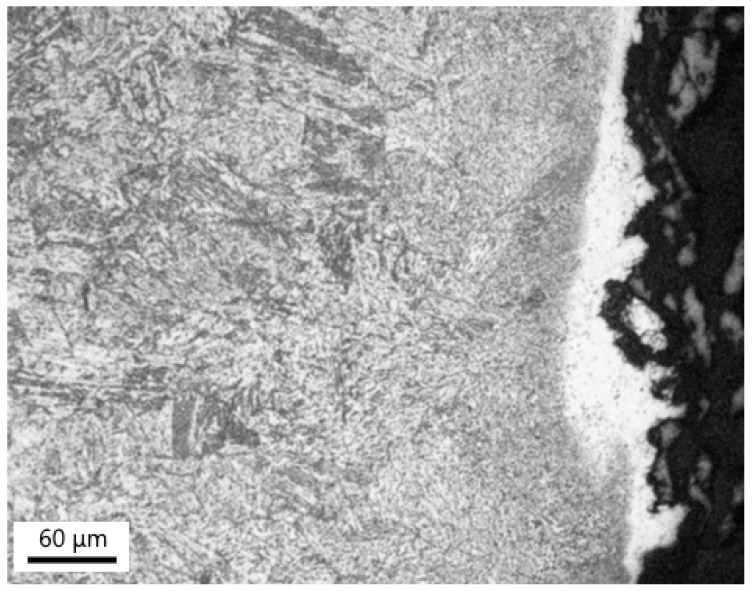
Fracture line in the partially decarburized CGHAZ of P23 steel close to the P23/WM91 fusion boundary—sample A2.

**Figure 10:**
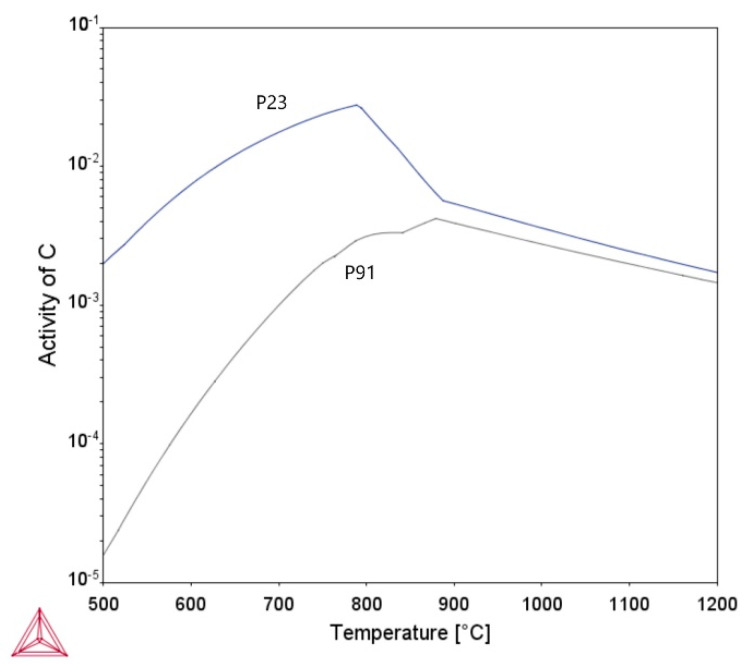
Temperature dependence of carbon activity in P23 and P91 steels, calculated using Thermo-calc software.

**Figure 11:**
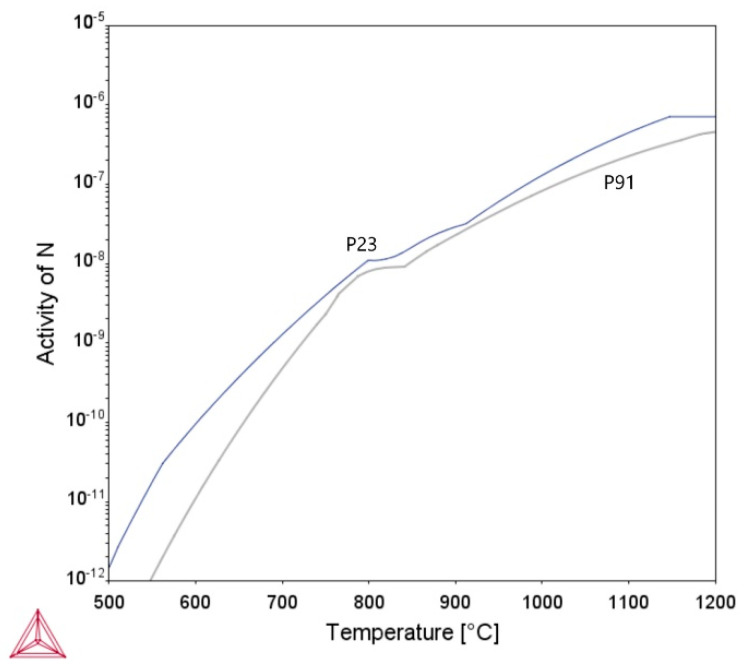
Temperature dependence of nitrogen activity in P23 and P91 steels, calculated using Thermo-calc software.

Figure 9 documents part of the fraction line in sample A2. It propagates in the partly decarburized GCHAZ of the P23 steel along the P23/WM91 fusion boundary. The driving force for carbon redistribution during PWHT and subsequent creep exposure is a function of differences in carbon activity across the fusion boundary. At temperatures below A_c1_, the carbon activity in the P23 steel is significantly higher than that of the P91 steel (Figure 10). This represents the driving force for the diffusion of carbon from P23 steel to P91 steel during the PWHT and subsequent creep testing. Carbon activity decreases with either decreasing carbon content in the steels or the presence of carbide-forming elements in the matrix. The differences in nitrogen activity between the two steels are much less significant, and therefore only an insignificant redistribution of nitrogen is anticipated (Figure 11).

**Figure 12:**
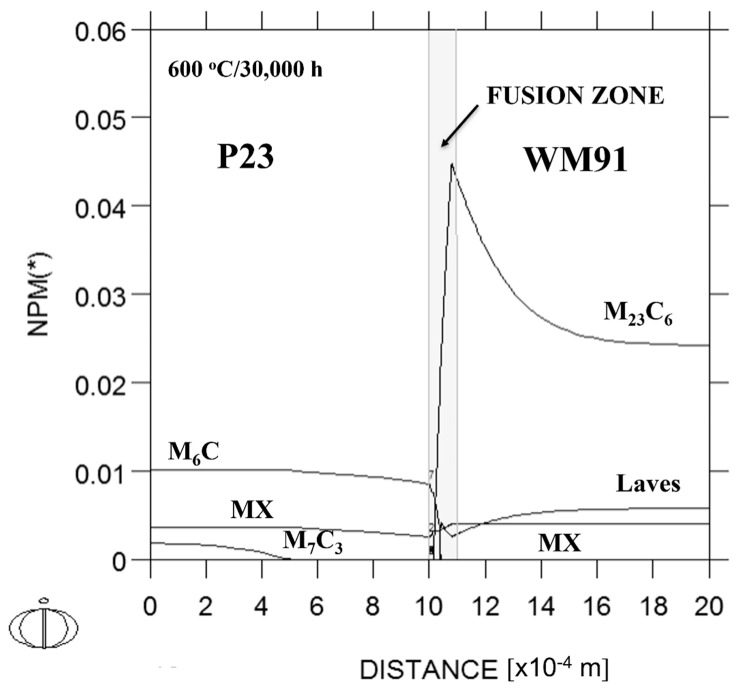
Kinetic simulation of minor phase profiles across the P23/P91interface, considering the fusion zone with a thickness of 0.1 mm, calculated using Dictra software. NPM(*) - mole fractions of individual phases present in the graph.

Figure 12 shows the results of the kinetic simulation of the minor phase profiles in the P23/P91 welds after thermal exposure at 600 °C/30,000 h, considering the fusion zone with a thickness of 0.1 mm. The minor phases M_6_X, MX and M_7_C_3_ are predicted to be thermodynamically stable minor phases in P23 steel. The results show that the partial decarburization of the CGHAZ in P23 steel is accompanied by a pronounced dissolution of M_7_C_3_ carbides. Partial decarburization is accompanied by an insignificant decrease in the MX and M_6_C fractions in the P23 steel close to the fusion boundary. A significant increase in the M_23_C_6_ fraction in the fusion zone suggests that carburization in this transient area with a linear gradient of chromium content is very intensive. The maximum fraction of the M_23_C_6_ phase is predicted near the end of the fusion zone. On the side of the P91 steel, the M_23_C_6_ fraction is expected to continuously decrease to a value corresponding to P91 steel. Carburizing the fusion zone and adjacent P91 steel has a negative effect on the fraction of the Fe_2_Mo Laves phase in the microstructure. The fraction of the MX phase does not appear to be affected.

**Figure 13:**
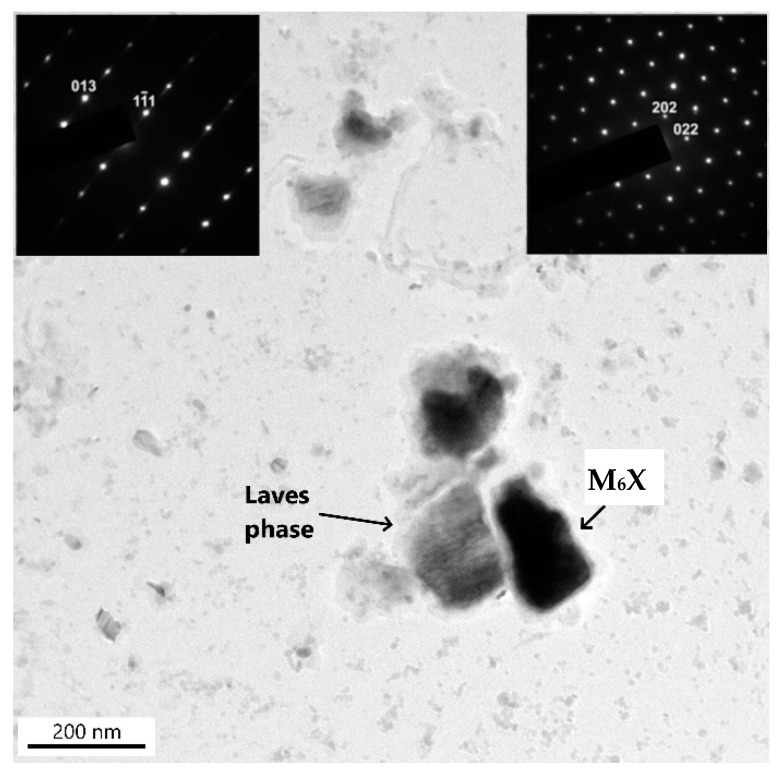
M_6_X and Fe_2_ (W, Mo) Laves-phase particles in the CGHAZ of P23 steel. Inserts: [$\bar{4}\bar{3}$1]_Laves_ and [11$\bar{1}$]_M6X_. Sample A2.

**Figure 14:**
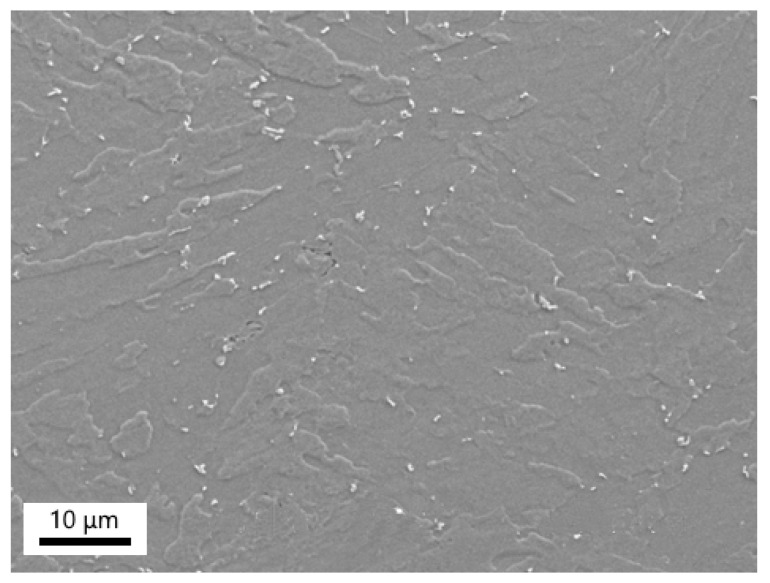
Distribution of M_6_X and Fe_2_ (W, Mo). Laves-phase particles in the CGHAZ of P23 steel—BSE image. Sample A2.

The experimental studies of precipitation in the partially decarburized CGHAZ of the base material P23 in sample A2 revealed that during long-term creep exposure, M_7_C_3_ and M_23_C_6_ completely dissolved (Figure 13). On the other hand, the MX and M_6_X particles in this part of the weld joint were preserved. The number density of the titanium-rich MX phase in the CGHAZ of the P23 steel was greater than that of the secondary MX particles rich in vanadium. Furthermore, a small number of particles rich in tungsten and iron were present. Electron diffraction analysis identified two minor phases: an M_6_C phase and a hexagonal Laves phase of Fe_2_W type (Figure 13). Planar defects on the basal plane of the Laves phase caused a significant streaking in spot diffraction patterns. The results of the EDX analyses of these two minor phases revealed that the chemical composition of both phases is very similar (Table 5). The particles of these phases could not be reliably discriminated in this way. The typical distribution of these minor phases rich in tungsten in the GCHAZ of the P23 steel of sample A2 is documented in the backscattered electron (BSE) image in Figure 14.

**Figure 15:**
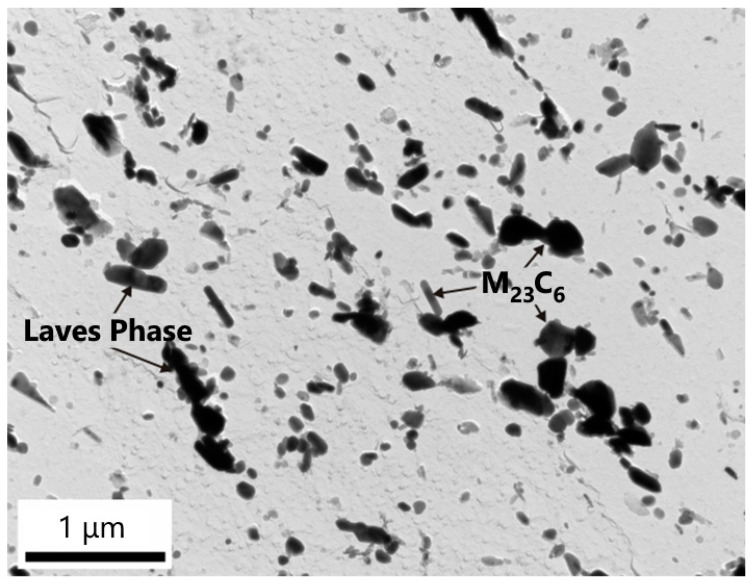
Precipitation in base material P91—sample A2.

The microstructure of the P91 steel after long-term creep exposure consisted of heavily tempered martensite. Figure 15 shows a typical distribution of precipitates in the base material P91 in sample A2. Coarser particles along the prior austenite grain boundaries and lath boundaries were identified as the Fe_2_Mo Laves phase and the M_23_C_6_ phase. Fine particles of the MX phase occurred in martensitic laths. Primary MX particles were rich in niobium; fine secondary MX particles contained niobium and vanadium. No modified Z-phase particles were present in the P91 steel of sample A2.

**Figure 16:**
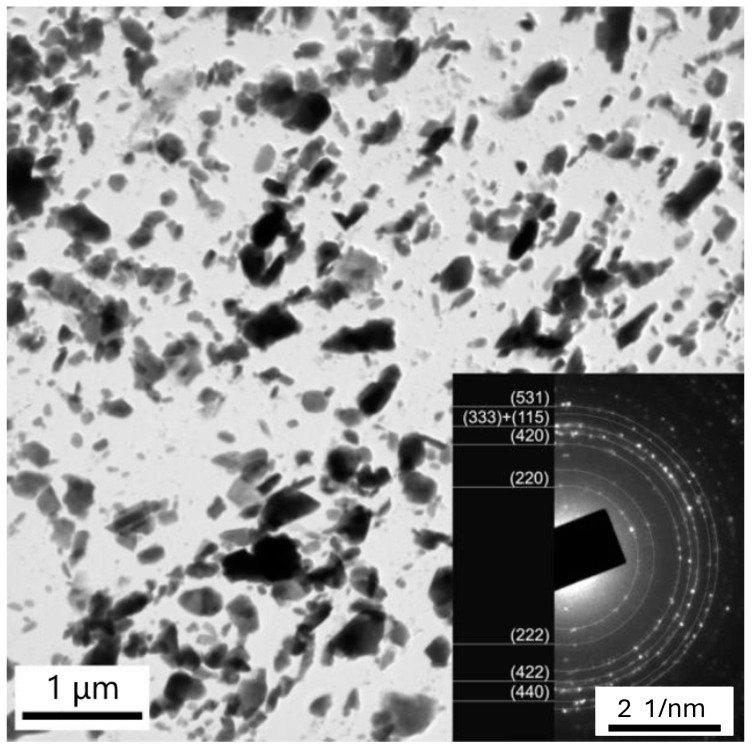
Heavy precipitation in the carburized fusion zone of P23/WM91. Insert: Ring diffraction pattern of the M_23_C_6_ phase. Sample A2.

The most intensive precipitation was observed in the P23/WM91 fusion zone (Figure 16). Particles of different shapes and sizes were present in this area. The SAED analysis proved that most particles were M_23_C_6_ carbides (see insert in Figure 16). The chromium content in the M_23_C_6_ particles in the carburized fusion zone varied continuously from the side of the P23 steel to WM91 (Table 6). This proves that the carbon redistribution mainly occurred in the fusion zone with a continuous chromium gradient. As predicted by the thermodynamic simulations, the more chromium in the matrix, the less iron was found in the M_23_C_6_ particles. A small number of M_6_X-phase particles coexisted in the carburized layer together with M_23_C_6_ particles. It can be expected that nitrogen redistribution may have contributed to the stabilization of this phase. No Fe_2_Mo Laves-phase particles were identified in the carburized area of sample A2.

**Figure 17:**
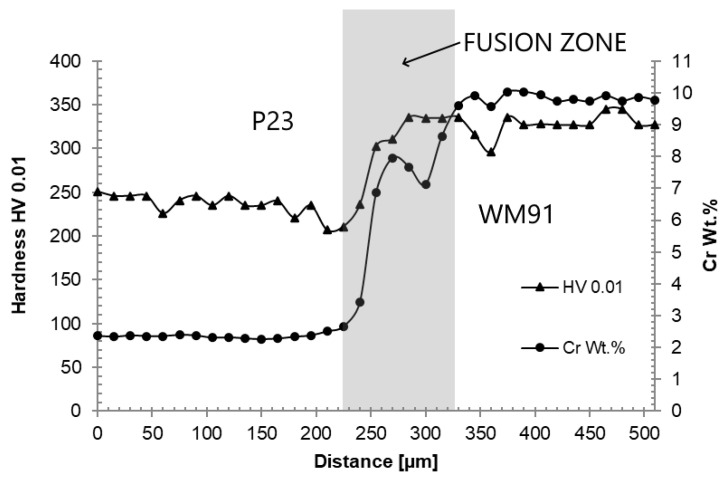
Microhardness (HV 0.01) and chromium gradients across the P23/WM91 fusion zone—sample A2.

Figure 17 shows the microhardness (HV 0.01) and chromium profiles across the P23/WM91 fusion zone in sample A2. The microhardness increase is closely related to the chromium gradient in the fusion zone.

### 3.3. Microstructure Characterization of Weld B

**Figure 18:**
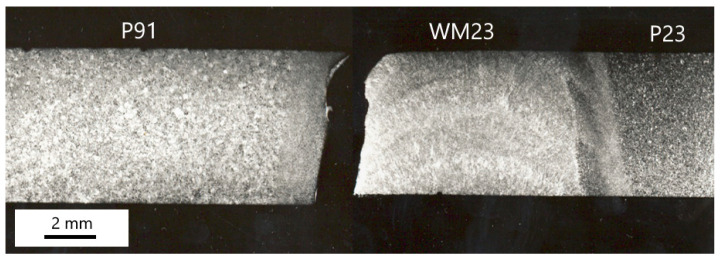
Longitudinal section of the ruptured sample B1.

Figure 18 shows a longitudinal section of sample B1. The creep fracture occurs in the partly decarburized zone of the weld metal WM23 close to the WM23/P91 fusion zone. As is evident in Figure 5, this type of creep failure was exclusive to all cross-weld samples tested at 550 and 600 °C. Similarly to Weld A, creep damage occurred simultaneously in different parts of the samples [32].

**Figure 19:**
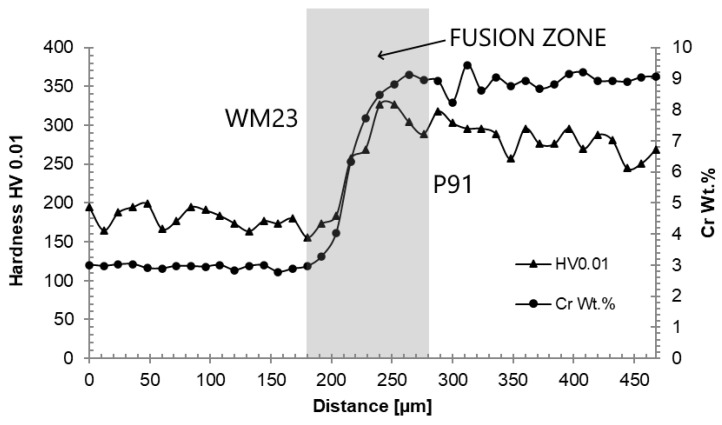
Microhardness (HV 0.01) and chromium profiles across the WM23/P91 interface—sample B1.

Figure 19 documents the microhardness and chromium gradients in the carburized WM23/P91 fusion zone in sample B1. The increase in microhardness is closely related to the chromium gradient in the fusion zone. The microhardness level of WM23 is lower than that of the P23 base material in Weld A. This is a consequence of the differences in chemical compositions, thermo-mechanical history, and microstructure evolution in both samples.

**Figure 20:**
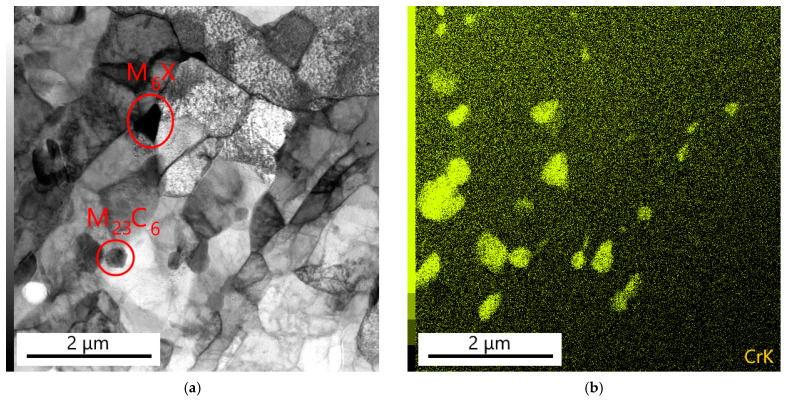

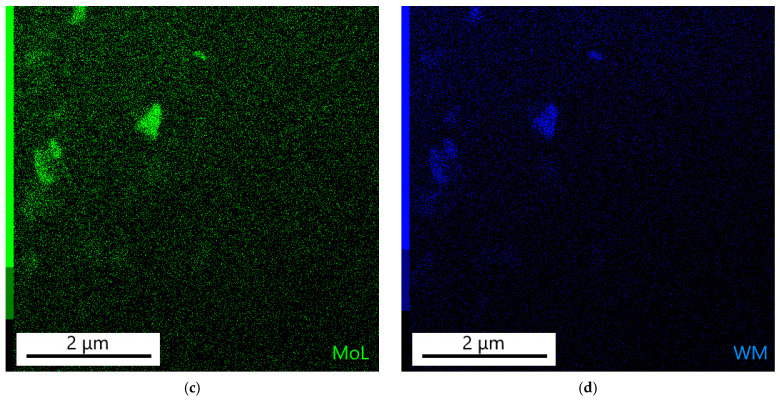
Precipitation in the WM23/P91 fusion zone (mainly M_23_C_6_ and some Mo- and W-rich M_6_X particles): (**a**) bright field; (**b**–**d**) X-ray maps of Cr, Mo and W. Sample B1.

The microstructural characterization of the WM23/P91 fusion zone was carried out with a TEM using thin foils prepared by the FIB technique [33]. Figure 20a–d show the substructure of sample B1 in the area containing approximately 3.5 wt.%Cr, which corresponds to the beginning of the fusion zone. Fine ferritic grains in the matrix proved that bainite recrystallization took place during long-term creep exposure at 550 °C. In the ferritic matrix, there are particles of minor phases. Detailed SAED analysis revealed that most of the particles corresponded to the chromium-rich M_23_C_6_ phase. The X-ray map of chromium in Figure 20b documents the distribution of particles of this minor phase. Furthermore, diffraction analysis revealed that some particles were formed by the molybdenum- and tungsten-rich M_6_X phase. The X-ray maps of tungsten and molybdenum in Figure 20c,d define the distribution of the particles of this phase.

**Figure 21:**
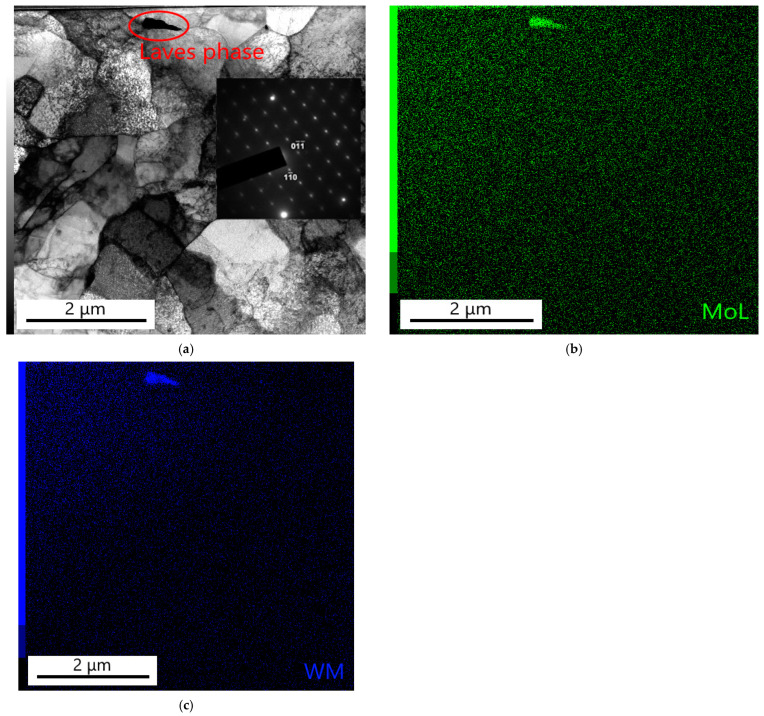
Partly decarburized zone of WM23: (**a**) bright field (insert: spot diffraction pattern of Fe_2_ (W, Mo) Laves phase with zone axis $[{11\bar{1}]}_{Laves}$); (**b**,**c**) X-ray maps of Mo and W. Sample B1.

Figure 21a–c show the substructure of the thin foil prepared in the partly decarburized zone of the P23 steel. The chromium content in the matrix, as determined by the EDX analysis, was about 2.5 wt.%. The matrix consists of recrystallized ferritic grains. The circle in Figure 21a marks the precipitate that was identified by SAED as the Fe_2_ (W, Mo) Laves phase (see insert). Figure 21b,c show molybdenum and tungsten X-ray maps. It is evident that in the field of interest, there is only one particle of the Fe_2_ (W, Mo) phase. No chromium-rich minor phases were observed. The chromium-rich carbides in this partially decarburized area are expected to dissolve during long-term creep exposure.

The Thyssen Cr2WV filler consumable did not contain titanium, and therefore no titanium-rich MX particles were present in the WM23 sample. The results of the detailed TEM analysis of the WM23/P91 fusion zone and the adjacent partially decarburized WM23 zone in Weld B prove that the microstructural evolution in these regions of interest was similar to that of Weld A. The results of the minor-phase identification in the base materials P23 and P91 in samples A2 and B1 were also identical.

## 4. Discussion

The results of the stress rupture tests on Welds A and B demonstrate that Weld A exhibits a better creep resistance than Weld B at all test temperatures. The long-term creep tests showed that the microstructure that was the weakest in terms of creep was associated with the partially decarburized CGHAZ of the P23 or WM23 steel. The selection of weld metals is normally undertaken to overmatch the room temperature strength of the base materials being welded. The higher creep resistance of Weld A can be related to the increased constraints that the stronger filler material provides. Generally, the use of a weaker filler material allows for more uniform stress distribution and has the effect of shortening the rupture time during creep rupture tests. The qualification testing of weldments is an important consideration for the selection of welding procedures, filler consumables and PWHT parameters. A key difference in the world’s construction codes is whether the use of Charpy impact toughness tests is required for an assessment of fracture resistance. In the case of welding P23 and P91 steel grades, a major supplier of ferritic filler consumables recommends using a filler metal matching P23 steel, because at room temperature its bainitic microstructure generally exhibits better Charpy impact values than martensitic filler materials [2]. This demonstrates that the constraints imposed by codes can influence important aspects of welding procedures.

**Figure 22:**
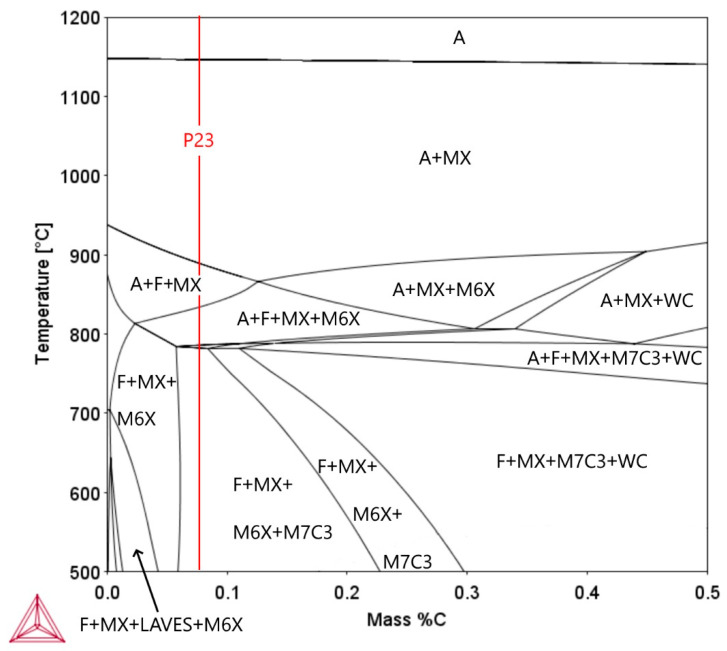
Effect of carbon content on equilibrium minor phases in P23 steel. The red line shows the carbon content in the heat investigated, calculated using Thermo-Calc software.

The results of the evaluation of the minor phases in the partially decarburized CGHAZ of P23 or WM23 steel after long-term creep exposure at 550 °C demonstrate that in both Weld A and Weld B, there are particles of the Fe_2_ (W, Mo) Laves phase. This minor phase has not been reported in previous publications dealing with precipitation processes in T/P23 steel [16,18]. Figure 22 shows the effect of carbon in P23 steel on minor phases that are thermodynamically stable.

At the nominal carbon content of P23 steel (red line), the thermodynamically stable phases predicted in the temperature interval of 500–600 °C include the MX, M_6_C and M_7_C_3_ phases. The partial decarburization of P23 steel is accompanied by the destabilization of M_7_C_3_ carbides, and at carbon contents lower than about 0.04 wt.%, the Fe_2_W Laves phase is predicted as the thermodynamically stable minor phase. This can be considered as an explanation for the presence of the Fe_2_ (W, Mo) Laves phase in the partially decarburized areas of the P23/P91 welds. However, the fraction of this phase in both samples investigated was low, and therefore the expected effect of this phase on the properties of the partially decarburized zone of P23 steel is insignificant.

Table 7 shows the results of the minor phase identification in the basic parts of sample A2. For P23 steel, Thermo-Calc simulations using the TCFE8 database predicted the following thermodynamically stable minor phases at a temperature of 550 °C: M_7_C_3_, M_6_X and MX. In addition to these minor phases, an experimental study confirmed the presence of the M_23_C_6_ phase. Precipitation studies on P23 steel after 500 °C/77,087 h creep exposure did not reveal the presence of this phase [32]. These results suggest that the M_23_C_6_ phase can be regarded as a metastable minor phase in P23 steel. The partial decarburization of P23 steel was accompanied by the dissolution of the thermodynamically less stable carbides of M_7_C_3_ and M_23_C_6_. The dissolution of these carbides provided free carbon atoms for diffusion across the fusion boundary.

TEM investigations confirmed that carburization mainly took place in the P23/P91 fusion zones (Figure 20). The gradient of the chromium content in the fusion zones is also associated with the gradient of carbon activity, which represents the driving force for carbon redistribution. Sopousek and Foret [34] reported that the carbon activity in the ferritic matrix at 600 °C decreased by at least one order, while gradually increasing chromium contents from 2.5 to 8.5 wt.%. The redistribution of carbon was accompanied by an increase in microhardness values in the fusion zones (Figure 17 and Figure 19).

The identified minor phases in the decarburized zone of P23 steel, the P23/WM91 fusion zone, WM91, and the P91 steel of sample A2 were identical to the predicted stable minor phases (Figure 12 and Table 7).

**Figure 23:**
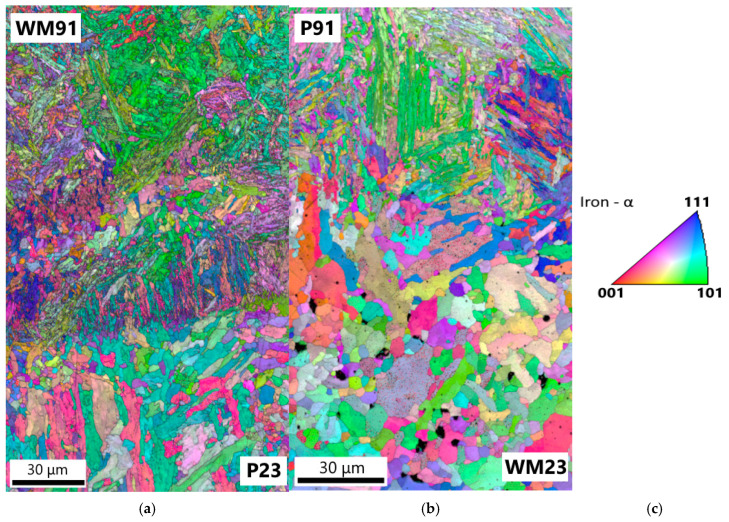
IPF (normal direction) + image quality maps across the fusion zones P23/WM91 and WM23/P91: (**a**) sample A2, Weld A; (**b**) sample B1, Weld B; (**c**) color coding.

Figure 23a–c show the results of the EBSD investigations of the samples of Welds A and B with similar creep exposure parameters. Figure 23a shows the inverse pole figure (IPF) orientation map of sample A2 in the P23/WM91 fusion zone. In the partially decarburized CGHAZ of the P23 steel, bainitic sheafs (platelets of bainitic ferrite) [35] are well defined up to the fusion boundary. However, differences in the shading of the color inside the individual platelets of the bainitic ferrite indicate that the recovery processes occurred in the fusion zone during long-term creep exposure at 550 °C. No fully recrystallized fine grains of ferrite were observed.

Figure 23b shows the IPF orientation map across the WM23/P91 fusion zone in sample B1. Small equiaxed ferritic grains are present in the partly decarburized layer of WM23 close to the WM23/P91 fusion zone. The platelets of bainitic ferrite are no longer evident in the weld metal. Dark globular particles in the weld metal represent the oxides formed during welding. The observed recrystallization of bainite in WM23 contributed to a lower hardness of WM23 compared to the base material of P23 steel. The demonstrated higher microstructural stability of the base material P23 compared to WM23 is consistent with the better creep resistance of Weld A.

The resistance of the bainitic/martensitic matrix to recrystallization is strongly affected by the thermodynamic and dimensional stability of minor-phase particles. Precipitates can pin grain boundaries and prevent recrystallization processes and grain growth. In the partially decarburized CGHAZ of Weld A and in the WM23 of Weld B in samples A2 and B1, particles of MX and M_6_X were present. Furthermore, some particles of the Fe_2_ (W, Mo) Laves phase precipitated in these areas. The thermodynamic stability of the MX particles in the P23 base material was increased due to a small addition of titanium in the studied melt (Table 1). Titanium-rich MX particles were more stable than vanadium-rich MX particles. On the other hand, WM23 did not contain titanium. Differences in the chemical composition of the partially decarburized areas of Welds A and B and thermal history differences affected the number density of the precipitates in the partially decarburized areas. More precipitates in the partially decarburized CGHAZ of the P23 steel in sample A2 contributed to a higher recrystallization resistance of Weld A.

## 5. Conclusions

1. Stress rupture tests of heterogeneous P23/P91 welds at 500, 550 and 600 °C revealed that the creep resistance of Weld A with a filler consumable matching P91 steel surpassed that of Weld B with a weld metal matching P23 steel. The extrapolated values of R_u/100,000h/T_ are higher for Weld A at all temperatures studied.
2. The failure locations were both stress- and temperature-dependent. The failure locations of most cross-weld samples that ruptured at 550 and 600 °C corresponded to the partially decarburized area of P23 steel or WM23 close to the fusion boundary. This is related to the redistribution of carbon across the fusion boundary. At temperatures of 500 and 550 °C and high applied stresses, failures occurred in the ICHAZ of the P23 steel.
3. The partial decarburization of the CGHAZ in the P23 steel (or WM23) was accompanied by the dissolution of M_7_C_3_ and M_23_C_6_ particles. The precipitates present in these areas included those of the MX, M_6_X and Fe_2_ (W, Mo) Laves phases. Thermodynamic simulations proved that the precipitation of the Fe_2_ (W, Mo) phase in the CGHAZ of the P23 steel and in the partially decarburized WM23 was a consequence of partial decarburization in these areas. This minor phase does not precipitate in the P23 base material/weld metal with a nominal carbon content.
4. Thermodynamic and kinetic simulations of carbon redistribution and minor-phase evolution in the P23/P91 welds across the fusion line predicted that carburization occurs primarily in the fusion zones of dissimilar metals. The experimental findings proved these results. The dominant minor phase in the carburized zones was M_23_C_6_ carbide.
5. Weld A was more resistant to recovery/recrystallization processes in the partially decarburized area of the CG HAZ in the P23 steel than Weld B in the partially decarburized zone of WM23, close to the WM23/P91 fusion zone.
6. These results confirm that the higher creep resistance of Weld A is related to its higher microstructural stability.

## Figures and Tables

**Table 1:** Chemical compositions of the base materials and weld metals, wt.% [28].

Material	C	S	Mn	Si	P	Cu	Ni	Cr	Mo	V	Ti	Nb	W	N	Al
P23	0.08	0.006	0.55	0.27	0.009	0.04	0.08	2.11	0.07	0.23	0.06	0.01	1.70	0.013	0.012
WM23	0.07	0.008	0.44	0.20	-	0.04	0.17	2.47	0.06	0.25	-	0.02	1.62	0.018	-
P91	0.11	0.004	0.51	0.38	0.015	0.17	0.42	8.67	1.00	0.23	0.01	0.07	0.01	0.048	0.012
WM91	0.11	0.008	0.66	0.21	0.009	0.04	0.82	9.50	1.02	0.22	0.01	0.04	0.06	0.028	-

**Table 2:** Creep parameters of the studied samples.

Samples	Temperature [°C]	Stress [MPa]	Time to Rupture [h]	Reduction in Area [%]
A1	600	90	5171	8.4
A2	550	115	26,386	8.2
B1	550	100	32,669	8.8

**Table 3:** Calculated and standardized creep rupture strength R_u/100,000h/T_ values for heterogeneous welds P23/P91 at 500, 550 and 600 °C.

T [°C]	Weld A R_u/100,000h/T_ [MPa]	Weld B R_u/100,000h/T_ [MPa]	Mean P23 (EN10 216-2 [31])	Low P23 (EN10 216-2 [31])
500	167	137	206	165
550	93	76	145	116
600	38	34	79	63

**Table 4:** Chemical compositions of minor phases in BM P23, wt.%—sample A2.

Phase	V	Cr	Mn	Fe	Mo	W
M_7_C_3_	4.7 ± 0.3	46.8 ± 1.4	2.7 ± 0.2	37.0 ± 1.5	N.A.	8.8 ± 1.8
M_23_C_6_	1.6 ± 0.5	28.0 ± 2.3	3.7 ± 0.2	44.7 ± 1.4	N.A.	22.0 ± 1.1
M_6_X	1.8 ± 1.0	4.6 ± 0.6	2.4 ± 0.3	16.7 ± 1.2	4.9 ± 0.7	72.0 ± 1.2

Note: The results represent arithmetic means and corresponding standard deviations. N.A.—not analyzed.

**Table 5:** Chemical compositions of the M_6_X and Fe_2_ (W, Mo) Laves-phase particles in the partly decarburized CGHAZ of P23 steel, wt.%—sample A2.

Phase	V	Cr	Fe	Mo	W
M_6_X	1.2 ± 0.6	3.1 ± 0.8	28.4 ± 5.9	6.9 ± 1.2	60.5 ± 5.2
Fe_2_ (W, Mo)	-	2.9 ± 0.5	32.9 ± 3.7	5.7 ± 0.8	58.5 ± 3.4

Note: The results represent arithmetic means and corresponding standard deviations.

**Table 6:** Chemical compositions of M_23_C_6_ particles in the carburized zone, wt.%—sample A2.

Location	V	Cr	Mn	Fe	Ni	Mo	W
On the side of P23	1.2 ± 0.2	50.6 ± 3.1	2.6 ± 0.3	26.4 ± 3.8	-	8.3 ± 1.1	10.9 ± 2.1
On the side of WM91	0.7 ± 0.1	65.9 ± 1.0	1.7 ± 0.2	15.2 ± 0.3	1.1 ± 0.1	14.7 ± 1.0	0.7 ± 0.1

Note: The results represent arithmetic means and corresponding standard deviations.

**Table 7:** Results of the minor phase identification in sample A2, Weld A.

Area	Minor Phases
BM P23	M_7_C_3_, M_23_C_6_, M_6_X, MX
P23 decarb. zone	MX, M_6_X, Laves phase Fe_2_(W,Mo)
P23/WM91 fusion zone	M_23_C_6_, MX, M_6_X
WM91	M_23_C_6_, NbX, secondary MX, Laves phase Fe_2_Mo
BM P91	M_23_C_6_, NbX, secondary MX, Laves phase Fe_2_Mo

## Data Availability

Data from this article can be found online at https://doi.org/10.5281/zenodo.14238779 (accessed on 28 November 2024).
